# Platforms for High-Throughput Screening and Force Measurements on Fungi and Oomycetes

**DOI:** 10.3390/mi12060639

**Published:** 2021-05-30

**Authors:** Yiling Sun, Ayelen Tayagui, Sarah Sale, Debolina Sarkar, Volker Nock, Ashley Garrill

**Affiliations:** 1 Biomolecular Interaction Centre, Department of Electrical and Computer Engineering, University of Canterbury, Christchurch 8041, New Zealand; yiling.sun@canterbury.ac.nz (Y.S.); ayelen.tayagui@canterbury.ac.nz (A.T.); sarah.sale@pg.canterbury.ac.nz (S.S.); debolina.sarkar@pg.canterbury.ac.nz (D.S.); 2 The MacDiarmid Institute for Advanced Materials and Nanotechnology, Wellington 6140, New Zealand; 3 School of Biological Sciences, University of Canterbury, Christchurch 8041, New Zealand

**Keywords:** fungi, oomycetes, lab-on-a-chip, high-throughput screening, force measurement

## Abstract

Pathogenic fungi and oomycetes give rise to a significant number of animal and plant diseases. While the spread of these pathogenic microorganisms is increasing globally, emerging resistance to antifungal drugs is making associated diseases more difficult to treat. High-throughput screening (HTS) and new developments in lab-on-a-chip (LOC) platforms promise to aid the discovery of urgently required new control strategies and anti-fungal/oomycete drugs. In this review, we summarize existing HTS and emergent LOC approaches in the context of infection strategies and invasive growth exhibited by these microorganisms. To aid this, we introduce key biological aspects and review existing HTS platforms based on both conventional and LOC techniques. We then provide an in-depth discussion of more specialized LOC platforms for force measurements on hyphae and to study electro- and chemotaxis in spores, approaches which have the potential to aid the discovery of alternative drug targets on future HTS platforms. Finally, we conclude with a brief discussion of the technical developments required to improve the uptake of these platforms into the general laboratory environment.

## 1. Introduction

Fungi and oomycetes are morphologically similar, yet phylogenetically very distant, groups of microorganisms. Both play a key role in ecosystems, breaking down and recycling organic nutrients, and as such are essential players in the complex web of life on this planet. Certain species, however, are pathogenic and cause disease and death in plants and animals, which can have significant impacts on ecosystems, biodiversity, and biosecurity. Human activity and global climate change are contributing to the spread of these pathogenic species, a trend that will only increase in the future [1,2]. This is compounded by the fact that fungal and oomycete diseases can be difficult to treat, with increasing evidence of resistance to antifungal drugs [3,4]. To tackle these issues it is important to, firstly understand the mechanisms that underlie their pathogenic growth and, secondly, to be able to develop control strategies to prevent disease. There has been an increase in the use of microfluidic technology in recent years with the development of methods and platforms that promise to assist in this process.

In this review, we focus on existing high-throughput screening (HTS) to develop potential anti-fungal drugs and recent progress in force measurements on fungi and oomycetes, used to better understand the infective process and potentially inform future drug discovery. First, we briefly introduce the infection strategies of pathogenic fungi and oomycetes, and the mechanisms underlying their invasive growth. We then summarize existing HTS platforms based on both, conventional and Lab-on-a-Chip (LOC) techniques, which predominantly focus on analyzing hyphal growth parameters to test the potential of anti-fungal/oomycete agents. The LOC approaches, in particular, extend HTS to the isolation of individual cells at specific stages of the fungal/oomycete life cycle, and we cover the methodologies that can be used to enable these including microfluidic droplets, hydrodynamic flow-assisted manipulation and pneumatic microvalves. This is followed by a more specialized review of methods that have previously been used for force measurements on hyphae and other organisms, such as optical tweezers and strain gauges, and then cover the more recent on-chip approaches using LOC techniques. To penetrate through host tissue, hyphae and invasive structures are thought to secrete wall digesting enzymes and to generate protrusive forces that enable them to act as pressurized drill bits [5,6,7,8,9]. Finally, we briefly introduce emerging LOCs related to the study of electro- and chemotaxis in spores, processes that aid motile spores in finding plant targets and suitable infection sites.

These latter topics are of particular importance as force generation mechanisms and spore sensing of plant targets may present alternative targets for anti-fungal/oomycete drug development on future HTS platforms.

## 2. Pathogenic Fungi and Oomycetes

### 2.1. Infection Strategies

Fungi and oomycetes, when observed through a microscope, appear very similar even though in evolutionary terms these groups of organisms are very distant. Fungi are more closely related to animals than they are to oomycetes (which in turn are more closely related to diatoms, brown algae, and protists) [10]. Their similarity is an example of convergent evolution, where need/function (i.e., the necessity to source and obtain nutrients) has given rise to a particular structure (i.e., tubular-shaped cells called hyphae). Hyphae are the dominant vegetative cells of fungi and oomycetes and these extend by a process called tip growth [11]. This mode of growth enables them to find nutrients, and many are saprotrophs-organisms that “eat” dead or decaying organic materials (e.g., fallen leaves or wood on a forest floor) which get broken down externally and then taken up into the hyphae. Tip growth generates cells with a large surface area to volume ratio which ensures optimal nutrient uptake. As such, the fungi and oomycetes play a key role in maintaining the healthy balance of ecosystems by recycling nutrients.

Some fungal and oomycete species obtain their nutrients, not from organic detritus but living organisms and in doing so can cause disease. The most well documented of these is most likely the late blight of potatoes caused by the oomycete *Phytophthora infestans*, which devastated potato crops in the late 18th century [12]. Currently, there are numerous diseases that are affecting crops, biodiversity, and ecosystems, and these are set to increase and become more problematic, with human activity and climate change hastening their spread [1]. To obtain their nutrients from living material the pathogens must invade/enter the plant/animal. In these instances, hyphae and associated infection structures act as pressurized drill bits boring their way into host tissue [5,7]. 

**Figure 1:**
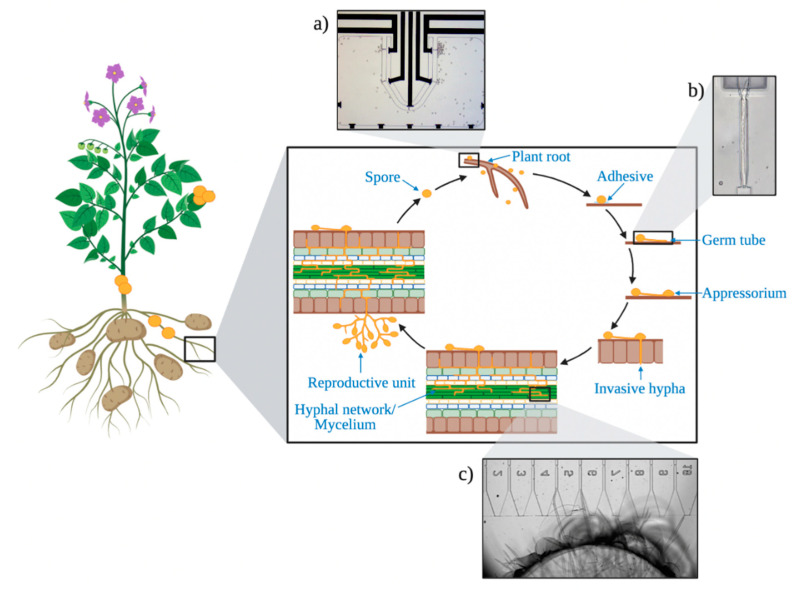
The roots, stem, and leaves of plants are susceptible to attack by fungal and oomycete pathogens. This example shows a simplified schematic of the invasive cycle of a root pathogen, including (clockwise, top to bottom) spores navigating a target root, attaching to the surface, germinating, and extending a germ tube, the formation of an appressorium, the penetration of the root tissue, and the formation of a mycelial network and reproductive structures. The reproductive structures then form more spores that can infect nearby plants. The insets show examples of existing microfluidic platforms that have been devised to study the respective stages of the invasion cycle. These are (**a**) the electrotactic/chemotactic attraction of individual zoospores to a plant root, (**b**) the protrusive force generated by a germ tube emerging from an individual zoospore and (**c**) the protrusive force generated by mature invasive hyphae.

Reproduction of fungi and oomycetes is very complicated and can occur both sexually and/or asexually depending on the species. In pathogenic species, the production of reproductive structures such as spores and conidia are important in the spread of disease and for successful colonization of a new environment [13]. In a very generalized scheme for an oomycete (See Figure 1), these structures can source new plant material by sensing electrical (electrotactic) or chemical (chemotactic) signals generated by the plant. The spores can then secrete adhesive material to firmly attach themselves to a plant surface before penetration [14]. For penetration, germination of spores occurs, which includes swelling of the spores and initiation of polarized growth and the formation of germ tubes [15]. Resulting germ tubes grow across the plant surface, navigating chemotropically and/or thigmotropically towards a suitable penetration site. Following penetration of the epidermis, invasive hyphae grow intercellularly and take up nutrients from the host plants [16]. 

### 2.2. Invasive Growth of Hyphae

Invasive growth of hyphae is thought to occur due to a complex interplay of the secretion of lytic enzymes, that are likely to reduce host tissue resistance and the application of mechanical force, which is generated by internal hydrostatic pressure (turgor) [17]. Turgor is the driving force for tip growth, as the cell wall at the very tip of the cell is plastic and will yield to the pressure, whereas further back the wall is elastic and will not yield to the pressure. This leads to anisotropic growth and the generation of tubular-shaped hyphae. Wall yielding at the tip will also lead to the generation of a protrusive force, thus enabling the hyphae to act as “pressurized drill bits” and the penetration of the host material [5,6,7,8,9]. In addition to modifications of turgor pressure and wall yielding, hyphae are thought to also regulate the protrusive force through modifications of the actin cytoskeleton [8,9].

## 3. High-Throughput Screening (HTS)

### 3.1. Conventional HTS

Given the significant diseases caused by fungi and oomycetes and the emergence of antifungal resistance, the efficient identification of new or alternative antifungal agents is of high priority. To this end, HTS of small molecules from libraries of synthetic compounds and natural products, originally developed for the pharmaceutical industry [18], has been used for antifungal drug discovery [19,20,21]. By applying HTS technology to the processes of phenotype-based and target-based drug discovery, approaches could be sped up and hit rates increased, owing to the screening of a multitude of small molecules [20]. In an example of this, Watamoto et al. tested a library of 1280 pharmacologically active compounds using HTS with antifungal susceptibility tests in 96 well-plates. They used this technique to identify antifungal agents with activity against the fungus *Candida albicans*, which is a commensal of humans, but which can cause significant disease in immunocompromised individuals [22]. Out of the 1280 compounds, only five compounds exhibited strong fungicidal effects, inhibiting the metabolic activity of *C. albicans* by over 90%, while only one compound, CV-3988, showed no cytotoxicity to human cells at a fungicidal concentration.

More recently, Lawrence et al. screened root and leaf extracts of four New Zealand native plants likely to produce anti-*Phytophthora* compounds [23]. Crude extracts were screened to evaluate their effects on zoospore motility, zoospore germination, and mycelial growth of the oomycetes *Phytophthora agathidicida* and *Phytophthora cinnamomi*. Flavanones, which had not previously been reported as anti-*Phytophthora* compounds, were purified from the kānuka leaf and demonstrated to have identical effects on these three lifecycle stages.

While being promising, the natural limitations of HTS in relation to fungal assays have so far led to the discovery of only a limited number of new compounds. In the HTS that have been reported to date, fungi and selected compounds were cultured and screened in welled plates, but these studies have been hampered by unequal spore concentrations in each well, difficulties with spore immobilization and observation of their germination. More importantly though, being predominantly well-based, HTS approaches have been difficult to combine with other methods aimed at understanding possible alternatives and under-examined angles of attack for antifungal agents, such as the protrusive force generation during hyphal invasion.

### 3.2. Lab-on-a-Chip (LOC) Platforms for Tip-Growing Organism Screening

In recent years, several microelectromechanical systems (MEMS) and LOC technologies have emerged that assist in the study of the biomechanics of tip-growing cells and organisms, such as plant pollen tubes, filamentous fungi, and root hairs [24,25,26,27], as summarized in Table 1. In general, microfluidic platforms provide several advantages over traditional culture and observation techniques when applied to microorganisms. Due to their design, these platforms enable stable, long-term culture, co-culture, and compartmentalization in well-defined microenvironments, facilitating high-resolution observation, analysis and increased throughput, and allowing for precise manipulation and observation at cellular and multi-cellular levels [26,27].

#### 3.2.1. Hyphal Growth and Spore Monitoring

**Figure 2:**
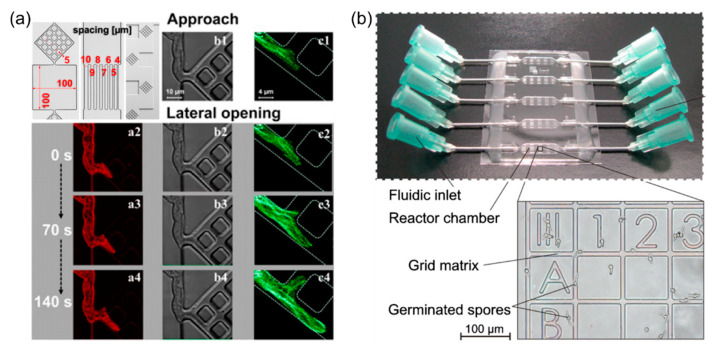
Growing hyphae and spore monitoring. (**a**) Microfluidic maze-like micro confined networks for analysis of hyphal space-searching mechanisms of fungi. Reproduced according to CC BY 4.0 from Held et al. [30]. (**b**) Microchamber array for screening of morphogenesis during spore germination and germ tube growth. Reproduced with permission from Demming et al. [34].

Platforms with three-dimensional microfluidic maze-like structures (see Figure 2a), mimicking aspects of the natural environment, have been deployed to evaluate the growth behavior and to analyze the space-searching growth mechanisms of fungal hyphae [28,29,30,31]. Observations of *Pycnoporus cinnabarinus* hyphae before and after they grew into microstructured areas showed that extension rates were not changed by confinement or obstacles, while hyphal branching rates were almost twice as high within the microstructures [28]. Differences in fungal growth and space exploration strategies between fungal species were found using various levels of containment and microstructures on polydimethylsiloxane (PDMS)-based chips [30,31]. Similarly, based on confinement structures on microfluidic chips, Baranger et al. grew hyphae of two species, *Talaromyces helices* and *Neurospora crassa* into parallel microchannels that had nutrient and water supply carefully controlled, and monitored their growth behavior [32]. The microchannels were designed to be narrower than the hyphal widths. The growth rates were observed to drop in microchannels compared to those on agar plates, and for *N. crassa*, growth was also observed to be limited by the reduced nutrient supply.

Several microfluidic platforms have been utilized for screening and monitoring morphogenesis during spore germination and germ tube growth [33,34,35,36,37]. Grunberger et al. utilized a cultivation device with time-lapse live-cell imaging to analyze spore swelling rates and hyphal/mycelium growth rates of the fungus *Penicillium chrysogenum* [33]. The chip was made by bonding a PDMS pad onto glass, which had the spore suspension in the center. The chamber dimensions increased with ongoing mycelium formation because of the elasticity of the PDMS. Similarly, Demming et al. fabricated a parallel microbioreactor with a grid structure for the evaluation of conidial germination of *Aspergillus ochraceus* under conditions of different pH and temperatures, as shown in Figure 2b [34]. They found that the germination rate of conidia was greatest in a pH 5.5 medium and a temperature range of 26 to 30 °C. Geng et al. used a cultivation platform, which enabled fine control over the microchamber array to compartmentalize each chamber or to replace media components at any experimental time point to study morphological transitions of the yeast *Yarrowia lipolytica* cells under nitrogen limitation [35]. With their devices, each microchamber was connected to a pair of serpentine main channels with integrated membrane valves for medium or oil infusion. Finally, at a sub-hyphal level, a 2D spiral microfluidic device was used by Lee et al. to monitor in real-time the nuclear dynamics in *N. crassa* [37]. Circadian changes of growth rates and circadian clock-gated nuclear divisions could be observed on this platform, owing to the confined spiral channel and long-term imaging ability. 

Devices have also been used to study the growth of fungi/oomycetes with other organisms/viruses. An adapted “Plant-on-a-chip” platform was reported by Parashar and Pandey, which was used to grow roots of the model plant *Arabidopsis thaliana*, in order to investigate root-pathogen interactions between *Arabidopsis* plants and the sugar beet nematode (SBN) or the oomycete *Phytophthora sojae* [38]. Physically, the platform consisted of a set of parallel microchannels with individual inlet/outlet ports for plant seedlings and the inoculation of pathogens. Motile zoospores swam randomly in the microchannels and then started to settle close to the root tip up to 2 h after inoculation. In other more integrated examples, microfluidic platforms were used to study the interactions of phages with fungal mycelia [39] and the defense response to spatially confined predation by a fungivorous nematode [40]. 

Although the use of LOC techniques in the above studies enabled the study of individual hyphae under defined environmental conditions, most of the studies introduced spore suspensions into a microchamber for cultivation. This made it difficult to control the distribution of spores inside the chamber and immobilize organisms during media exchanges or the addition of other liquid reagents.

**Figure 3:**
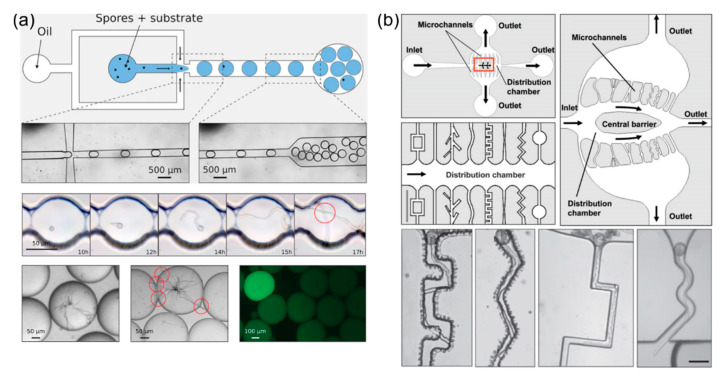
Single-cell compartmentalization and screening using Lab-on-a-Chip technology. (**a**) A high-throughput screening platform for large number of filamentous fungi, which combines droplet microfluidics with fluorescence-activated dielectrophoretic (DEP) sorting. Reproduced with permission from Beneyton et al. [43]. (**b**) TipChip-based long-term screening for pollen tube phenotyping using hydrodynamic flow-assisted manipulation. Reproduced with permission from Agudelo et al. [45].

In order to compartmentalize individual fungal or oomycete spores, droplet microfluidics have been used in conjunction with spore handling. Droplet microfluidics constitute a practical method for the encapsulation of single cells, allowing for chemical isolation and thus, quantitative control of reagent concentrations [41]. To phenotypically screen the anti-oomycete candidate chemical metalaxyl, Yang et al., encapsulated motile zoospores of the oomycete *Phytophthora sojae* and metalaxyl into microdroplets using a simple T-junction droplet generator platform [42]. Zoospore germination and germ tube growth at different chemical concentrations inside the droplets were tracked. Zoospores failed to germinate at metalaxyl concentrations greater than 2.0 mg/mL. Droplet microfluidics, in combination with fluorescence-activated dielectrophoretic (DEP) sorting, have also been used by Beneyton et al. in the development of an HTS platform for a large number of filamentous fungi (see Figure 3a) [43]. The spores of the fungus *Aspergillus niger* were encapsulated individually in 10 nL droplets, along with an α-amylase fluorogenic enzyme substrate. The platform was able to screen approximately 5 × 10^4^ droplets in 90 min and sort around 750 droplets of these (1.45%), corresponding to a 196-fold enrichment for those containing fungi. However, the average droplet volumes that were produced were too small for the long-term culture of the fungi. This meant that observations were restricted to the phases of spore germination or germ tube growth.

To address the problem of long-term screening, hydrodynamic flow-assisted manipulation has been used to trap individual spores on the platforms [44,45,46,47]. One particular challenge for the design of platforms for tip-growing cells is that firstly a spherical/oval spore needs to be trapped and then when it germinates a long cylindrical germ tube will emerge, necessitating a long growth channel next to the trap site. Agudelo et al. developed an experimental platform, named TipChip as shown in Figure 3b, enabling efficient and high-throughput large-scale phenotyping of pollen tubes that emerged from pollen grains [44,45]. The device allowed the suspended pollen grains to be transported through a distribution chamber, and to be positioned at the entrances of microchannels. Upon germination, emerging pollen tubes grew along the channels and could be exposed to a variety of mechanical obstacles and constraints. Once the pollen grains were trapped, the culture medium could be set to continuously flow in to maintain a nutrient-rich environment, while keeping grains trapped at the entrances of the microchannels by fluid pressure. Ghanbari et al. further improved the TipChip platform to achieve trapping of a single pollen grain at the entrance of each microchannel [46]. Improvements also guaranteed identical fluid flow conditions in all microchannels for more comparable pollen tube growth. The device was also modified to implement an on-chip bending test for the investigation of biomechanical properties of pollen tubes [47]. To achieve this, the growing pollen tubes were guided through a short microchannel and subsequently exposed to a bending force by the fluid flow orientated perpendicular to the tubes. 

#### 3.2.2. Single Cell Compartmentalization

While there are many advantages, one major intrinsic limitation of flow-assisted cell trapping is that the trapped cells must be constantly exposed to the fluid flow for immobilization. While this helps with nutrient supply, shear stresses applied to the cells pose the risk that trapped cells are mechanically compressed, which may alter biological responses, such as germination and cell growth [45,48,49]. On the other hand, if the applied fluid pressure is kept low to avoid potential shear stress related experimental artifacts, cells may escape during long-term culture, resulting in a relatively poor positioning or ultimately cell loss. Recently, several studies have reported parallelized manipulation and analysis of single tip-growing cells by applying single cell trapping and micro-valving methods [50,51].

**Figure 4:**
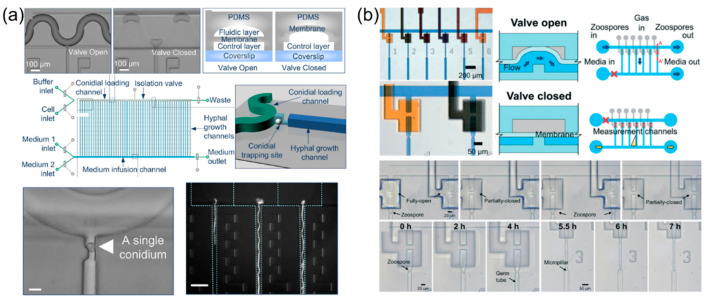
Single-cell compartmentalization and screening using pneumatic membrane valves. (**a**) A microfluidic channel array combined with a single normally-open microvalve, which enables compartmentalization of conidia after hydrodynamic trapping. Reproduced with permission from Geng et al. [50]. (**b**) A microfluidic platform using individually-controlled normally-closed microvalves to hydrodynamically trap and compartmentalize single zoospores. Reproduced with permission from Sun et al. [54].

A microfluidic channel array, as shown in Figure 4a, combined with a single pneumatic microvalve was employed to analyze hyphal growth of *N. crassa* conidia in various glucose concentrations and channel geometry conditions, nuclear migration, and gene expression [50]. Confined shallow channels in the trapping site were designed, which allowed only a single hypha to grow into a channel, even if several conidia accumulated at one site. The single normally-open microvalve located on a serpentine cell loading channel with a rounded cross-section was utilized to compartmentalize conidia after hydrodynamic trapping. Germ tubes that emerged from each conidium were prevented from extending into the cell loading channel by this valve, while compartmentalization allowed each to be exposed to equivalent environments. The extension rate of *N. crassa* hyphae did not show any significant difference in channels with widths ranging from 5 to 20 µm, although they generated numerous branches in the wider channels (12.5 to 20 µm). Nuclei were observed to retain their position relative to the tip as hyphae extended. While the device achieved a trapping efficiency of up to 70%, one drawback was the accumulation of multiple cells in single trapping sites. Hu et al. presented an adapted device for the characterization of mechanical properties of pollen tubes [51]. In this case, the pneumatic membrane valves were not only used to fix pollen grains in the trap, but also to exert a controlled uniform external pressure on the tubes acting as a form of soft indentation probe. The soft compression by the membrane produced a force that was distributed over a large area, allowing for the detection of averaged mechanical properties. The results indicated that the compressibility and stretch ratio increased considerably with an increase in the initial diameter of pollen tubes. This type of system has yet to be applied to study the biomechanics of fungal or oomycete hyphae.

More recently, we introduced a new microfluidic platform, which uses normally-closed membrane valves to enable the long-term hydrodynamic trapping and hermetic fluidic compartmentalization of single zoospores [52,53,54]. Each measurement channel, branched from the spore loading channel, contained a normally-closed valve and a horizontal constriction as a trap (See Figure 4b). This enabled the individual control of closing off each channel once a single zoospore was trapped. Partial closure of microvalves was also used until all the measurement channels had each trapped a single zoospore, to secure and improve the efficiency of single zoospore capture, as the flow in the measurement channels was not fully interrupted after one zoospore was captured. A 76.5% capture-to-growth success ratio was obtained using *A. bisexualis* zoospores and three different types of germination were observed during the on-chip culture. Through the use of parallel traps and measurement channels on the same device, the platform enabled HTS of zoospore germination and force measurements on the resulting germ tubes.

#### 3.2.3. Transport, Long-Term Culture, and Ease-of-Use of LOC Platforms

In parallel to the organism-specific developments discussed above, a variety of efforts have aimed to improve the usability issues to increase the uptake of these platforms for general use. These efforts have focused on improving the transport of devices and long-term culture on them to enable point-of-use organism analysis and better incorporation into traditional platforms used in biology, such as agar plates for testing/screening [55,56]. Millet et al. described a streamlined assembly, sterilization and self-priming of devices to increase access to microfluidics for studying fungi and other branched biological structures [56]. Three microfluidic platforms were shown enabling pre-priming, filling, and shipping of hydrated devices, with or without cultures. As a proof of concept, the fungus *Laccaria bicolor* was inoculated prior to sealing and kept growing during shipment. Transgenic bacteria, *Pseudomonas fluorescens*, could then be introduced and observed with high-resolution imaging for the study of bacterial-fungal interactions by collaborators. Additionally, *A. thaliana* seeds have been vacuum packaged on chips for 14 days of storage to test packaging influences on the seed germination process. One hundred percent of the seeds germinated within three days after opening the package and filling it with culture medium, and the growth rates showed no difference from the non-vacuum control.

### 3.3. Summary

Compared to a conventional HTS platform, LOC technology facilitates the screening at the single hypha or spore level, especially for the high-resolution monitoring of morphogenesis. While cultivation microchamber platforms [33,34,36,37,38,39,40] are easy to fabricate and operate, they suffer from control issues of spore distribution inside the chambers and cell immobilization during media or reagent exchange. Microfluidic droplet techniques [42,43] address some of these problems and allow for high-throughput detection and screening. However, they are typically not capable of enabling long-term monitoring and integration with other analysis methods, such as mechanical properties and protrusive forces, for example. Therefore, platforms using hydrodynamic flow-assisted manipulation [44,45,46,47], particularly combined with pneumatic membrane valves [50,51,52,53,54], appear most promising for the long-term screening of tip-growing cells. These platforms allow spores or pollen grains to be securely trapped and immobilized in designed trap-sites and the resulting germ tubes or pollen tubes can grow through integrated measurement microchannels. Moreover, platforms with independently controlled microvalves guarantee single spore trapping and compartmentalization under low shear stresses. In principle, these platforms allow for truly single cell parallelized screening of cellular heterogeneity, nuclear distribution and dynamics of fungi and oomycetes in response to exposure to various biocontrol strategies, albeit at the expense of more complex external flow control setups [54]. 

## 4. Protrusive Force Measurement and Electro-/Chemotaxis

### 4.1. Conventional Measurement of Protrusive Force

As discussed in the introduction, the ability to generate protrusive force plays a key role in the invasion process of fungal and oomycete pathogens. Despite this, we still have much to learn about the underlying biochemical and biomechanical mechanisms contributing to force generation. This is of particular importance, as force generation mechanisms may present an alternative target for new anti-fungal/oomycete drug development. 

One of the simplest methods to measure protrusive force has been to grow fungi and oomycetes on substrates with a range of known surface hardnesses, such as agar medium with yeasts [57] and non-biodegradable Mylar membranes [58]. Observation of agar penetration has, for example, demonstrated that an increase in agar medium from 2 to 8% *w*/*v* slowed down the rate of substrate invasion by the human pathogen *Wangiella dermatitidis* [57]. However, the application of this method remains limited by the relatively low medium gel strength, since even the hardest agar at 8% only exhibits a mechanical strength of around 0.1 µN/μm^2^.

To circumvent the reliance on the substrate properties, optical tweezer techniques have been used to measure the growth forces of hyphae [59]. With this technique, force measurement is facilitated by the placement of optically-trapped beads in front of the hyphal tips. Since the trapping forces are known, hyphal forces can be deduced from the translocation of the beads. The trapping force increases linearly with increasing incident laser power and can be estimated by dragging a bead at an increasing velocity until it escapes from the trap. However, even at the highest output laser powers (~70 mW), a hypha of the fungus *Neurospora crassa* was able to push a 4 µm bead out of a trap. This led to the conclusion that the growth forces of the hyphae analyzed were in the pico-Newton range. When similar experiments were carried out on a germ tube, the same-sized bead was moved forward but was not expelled out of the trap [60]. The tip of the germ tube was observed to swell and stop growing, with the growth restarting again after the removal of the bead. Based on this observation, it was concluded that the growth force generated by a germ tube is much smaller than that of a leading hypha. However, similar to the agar penetration method, the use of this technique has been limited to only a few species of fungi and oomycetes. It is also notable that the intense laser light required for measurements has also been shown to affect hyphal growth and thus potentially skew experimental results [60,61].

Another method that has been used to measure forces generated by hyphae utilizes miniature silicon bridge strain gauges [62,63,64]. In these studies, strain gauges were manually positioned with a motorized micromanipulator in a liquid-filled well close to leading hyphae. Forces were measured in proportion to the deformation of the silicon beam with an electrical-resistive element when hyphae grew from the solid medium into the liquid broth and then pushed against the strain gauge. The measurements from species belonging to various fungal taxonomic groups (Basidiomycota, Ascomycota, Zygomycota, and Chytridiomycota) showed that species with larger diameter hyphae were able to exert greater forces than those with smaller diameters [63]. However, no significant difference between species was observed with respect to the applied pressure, and the force generated, per unit area, ranged from 0.04 (*Sordaria*) to 0.09 MPa (*N. crassa*). Oomycete hyphae generated similar pressures from 0.01 to 0.17 MPa, which corresponded to a maximum of 54% of their turgor pressure. Measurement of forces exerted by the oomycete *A. bisexualis* revealed that the fluctuations in force had similar periodicity as that of extension rates of hyphae growing in agar medium [64]. However, the strain gauge technique risks underestimated the forces exerted by invasive hyphae, because these must emerge from solid media into the liquid broth before they can displace the transducer. In addition, it was observed that their tips change shape when they push silicon beams, which typically have a flat surface [7]. Finally, and most importantly, experiments with strain gauges can be technically challenging as the silicon beams of the gauges are typically 0.1 mm in thickness and 10 mm in width, while the diameter of hyphal tips is in the tens of micrometers range at the most. As a result of these limitations, and due to advances in microfabrication and device integration, researchers have started to investigate force generation utilizing methodologies based on microfluidics and LOC technology.

### 4.2. Microfluidic Platforms for Force Sensing

**Figure 5:**
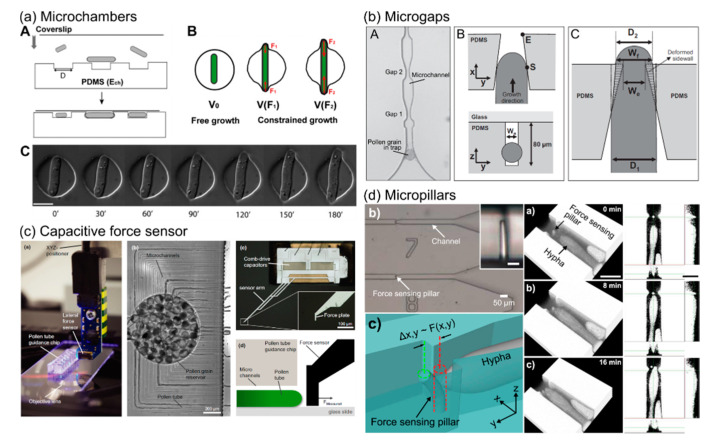
Force measurements on tip-growing cells. (**a**) A PDMS cylindrical microchamber array, employed as a force sensor to measure the extension mechanics of single cell fission yeast. Reproduced with permission from Minc et al. [65]. (**b**) A microfluidic device, containing microchannels, each with a sequential array of four narrowing gaps, to measure penetrative forces exerted by pollen tubes. Reproduced according to PNAS License to Publish from Nezhad et al. [66]. (**c**) MEMS-based capacitive force sensor, with sensor arm and force plate on the front-end, to quantify the force exerted by pollen tubes. Reproduced with permission from Burri et al. [67]. (**d**) An elastomeric micropillar platform containing single micropillars in channel constrictions, which enable the simultaneous measurement of hyphal growth and protrusive forces exerted by individual fungal hyphae. Reproduced with permission from Sun et al. [73].

One of the first uses of LOC technology to study forces in fungi was the pioneering use of a PDMS array, which contained 10,000 cylindrical microchambers, which acted as force sensors to measure the extension mechanics of the fission yeast *Schizosaccaromyces pombe* [65]. Yeasts are single-cell fungi that can be ellipsoid or rod-shaped. The elasticity (Young’s modulus) of the PDMS microchambers ranged from 0.1 to 1.5 MPa. Single rod-shaped fission yeast cells were introduced into chambers, which were longer than the seeded cells, and the cells were left to extend until they pushed against the wall of chambers, as shown in Figure 5a. By analyzing the growth of the cells and the deformation of the chambers, an internal effective turgor pressure of 0.85 ± 0.15 MPa and the force-velocity relationships could be obtained. The microchambers were deformed from a round shape into an oval shape due to the increase in exerted force, resulting in a gradual decrease in the growth rates of the yeast cells. These microchambers may not be suitable for hyphal species due to the different morphologies of these compared to rod-shaped single yeast cells, and also the difficulties in seeding small single wells with fungi/oomycetes.

A system that is more applicable to hyphae would be that which has been used to study force generation in pollen tubes. Pollen tubes are the reproductive structures that grow out from pollen grains and which facilitate sexual reproduction in flowering plants. The tubes, similar to hyphae, are tubular-shaped cells that extend by the process of tip growth. In one such study, PDMS devices were developed to measure the penetrative forces exerted by the pollen tubes of *Camellia japonica* [66]. Pollen grains were transported through a distribution chamber and trapped at the entrances of microchannels (see Figure 5b). Pollen tubes were then guided to grow through the microchannels, which contained a sequential array of four narrowing gaps, ranging from 17 to 10 µm across. The cells applied a pushing force deforming the sidewalls of moderately-sized gaps (pollen tube diameter/gap size ratio ≤1.20) and constricted themselves when passing through narrower gaps (which had a ratio of between 1.20 and 1.33). The pressure, when the tube reached the narrowest region of the gap, was estimated to be 0.15 MPa using finite element methods. From this, the dilating force was estimated to be 14.7 µN at the moment of maximum contact. The results revealed that the pollen tubes regulated cell wall compliance to meet increasing mechanical impedance, resulting in a change in exerted force. However, the tapered shape of the microgaps and the interaction between the pollen tube and the sidewalls, resulting in a combination of deformation of the sidewalls and an apparent reduction in pollen tube diameter, meant finite element simulations rather than an analytical model had to be used to derive the applied force. Furthermore, the fact that forces were measured via the deflection of the sidewall of microscopic gaps means that, when used with fungi or oomycete, pushing forces, rather than penetrative forces, would be measured due to different contact points with the obstacles.

More recently, Burri et al. combined a LOC device with a MEMS-based capacitive force sensor to quantify the force exerted by pollen tubes [67]. The force-sensing plate of the sensor was scaled down to 50 × 50 µm, providing a high force resolution of 0.18 µN. Pollen grains were injected into the inner reservoir and the resulting pollen tubes were guided to grow along parallel microchannels, as shown in Figure 5c, to ensure perpendicular contact with the force-sensing plate placed at the channel exit. After the initial contact of the pollen tubes with the sensor plate, a sharp force increase was observed and the force was measured up to a mean value of 9.6 ± 1.6 µN for *Lilium longiflorum* and 3.0 ± 0.6 µN for *A. thaliana*. After that point, pollen tubes were observed to stop growing for a few seconds, adjusting the direction of tip growth to avoid obstacles and then they resumed growth. By placing the force-sensing plate close to the exit of the microchannels, gaps with various widths and lengths could be formed. It was observed that the narrower and longer the gap the pollen tube had to penetrate through, the larger the measured force, with a maximum of 85 µN.

Unfortunately, the throughput of the system described above was limited because the capacitive force sensor with sensor arm and force plate on the front-end was mounted on a piezoelectric xyz-positioner, and only one measurement could be made at a time, even though multiple pollen tubes grew simultaneously in the parallel microchannels. This, combined with the fact that pollen tubes had to exit the LOC and bridge a gap, means the invasive conditions (i.e., growing through semi-solid media) for fungi and oomycetes would be difficult to recreate in this system. Moreover, because the pollen grains were randomly loaded in the reservoir, the tubes grew in a disorderly manner into the microchannel, potentially influencing measurements in adjacent channels. Nonetheless, the work demonstrates that with continuing sensor miniaturization and improvements in MEMS techniques, experiments similar to the original silicon strain gauge work may yet have a place in the study of force generation. Ghanbari et al. also fabricated a micrometer-sized, calibrated cantilever on a LOC device for force measurement, based on the strain gauge principle [68]. The PDMS microcantilever was designed to be 400 µm long and 30 µm wide to achieve a dynamic range of cantilever deflection of between 1 and 15 µN. A curved notch was added to the microcantilever for trapping the pollen tube apex to prevent the pollen tube from changing its growth direction when encountering a mechanical obstacle. Although the integrated PDMS cantilever addressed some of the problems of the capacitive force sensor, there were issues with the complicated fabrication of the end-free cantilever and reduced force resolution. As with the previous example, while promising, this system has not yet been used with fungal or oomycete hyphae.

We have recently reported an elastomeric micropillar platform containing single micropillars in channel constrictions, which enables the high-resolution imaging of individual fungal and oomycete hyphae and the measurement of the protrusive forces that they exert, as shown in Figure 5d [69,70,71,72,73]. The platform uses PDMS-based force sensors that were originally developed for use with the nematode *Caenorhabditis elegans* and combines monolithic integration with simple read-out via microscopy [74,75,76,77]. Individual hyphae were guided to grow along the channels and finally contact with the high aspect-ratio force-sensing pillars. The protrusive force exerted by hyphae could then be estimated in both magnitude and direction via the micropillar deflection that was recorded by a microscope and a mechanical deflection model. Using this method, a maximum total force of 10 µN at the hyphal tip of oomycete *A. bisexualis* was observed. Furthermore, the elastomeric micropillar-based protrusive force sensing has subsequently been combined with the capture and immobilization of individual zoospores in separate valve-controlled traps, to measure the forces generated by individual germ tubes [54]. As described further below, *A. bisexualis* zoospores germinated on the devices and the resulting germ tubes grew down the channels towards single elastomeric micropillars. Tracking of pillar movement allowed for the measurement of microNewton range protrusive forces imparted by the tips of the germ tubes. The forces generated by the germ tubes are smaller than those exerted by mature hyphae. Although the resolution of image processing still needs to be improved, micropillar force sensing is a relatively technically simple approach to measure individual hyphae or germ tubes in parallel, compared with other methods. The use of the parallel channels on the chips also facilitates HTS and the observation of multiple individual hyphae in the same experiments.

### 4.3. Microfluidic Platforms for Studying Electro- and Chemo Tactic Responses

Many oomycetes are able to infect and cause disease in plants because of the motility of their flagellated zoospores, which are produced as part of the asexual part of their life cycle. Thus, at times of heavy rainfall, or in waterlogged soils, the zoospores are carried in the water and/or swim towards nearby plants. They have an ability to sense the roots of these plants, responding to chemical and electrical signals that likely result from wound exudate and the spatial distribution of membrane transport proteins, respectively. The exact nature of these sensing mechanisms is unknown but may involve membrane proteins such as ion channels [78]. An ability to inhibit these tactic responses may provide a means to prevent plant disease.

The electrotactic properties have previously been studied using a micro-electrotaxis chamber, constructed out of microscope slides containing a central chamber measuring 1 cm^2^ × 0.1 cm [79]. Platinum wire electrodes were wired to each end of the chamber and were used to generate electric fields of between 50 and 500 mV cm^−1^. Isolated zoospores were added in solution to the central chamber and their tactic response to the electric field could be measured. Intriguingly some oomycete species are attracted towards the anode, whereas others are attracted towards a cathode [79] and clearly this is an area where more research is warranted. We have begun to use LOC devices to better replicate the root microenvironment (Figure 1a). It is possible to fabricate a central chamber with root-like architecture and to which electric fields can be applied through the inclusion of gold microelectrodes. Channels alongside the electrodes may also allow the inclusion of chemoattractants and this may allow the simultaneous assessment of both chemotaxis and electrotaxis.

### 4.4. Perspectives

To this date, the protrusive forces generated by fungal and oomycete hyphae have mainly been measured using conventional agar penetration methods [57,58], optical tweezer techniques [59,60,61], and strain gauges [62,63,64]. However, the first two methods have been found to be limited by the range of forces that can be measured, either due to the low mechanical strength of the agar or the limited range of bead trapping forces. For strain gauges, experiments can be technically challenging because of the comparatively large silicon beams of the gauges, compared to the hyphal tip size. Of the few microfluidic platforms developed to measure penetrative forces exerted by pollen tubes, none have yet been adapted to fungi or oomycetes. Similarly, while experiments with the microgap technique [66] should be technically straightforward to implement, the simultaneous deformation of the sidewalls and reduction in pollen tube diameter meant finite element simulations were required to deduce force values. This added complexity may make this technique less accessible to biology laboratories. MEMS-based capacitive force sensors [67], on the other hand, were able to achieve high-resolution pollen tube force measurements, however, the throughput of the system remains limited due to the setup of the force sensor. In comparison, elastomeric micropillar platforms [52,53,54,69,70,71,72,73] have been demonstrated to be capable of parallelized force sensing on both mature hyphae and germ tubes of fungi and oomycetes. Combining a comparatively simple fabrication process with a sensor readout based on conventional optical microscopy and analysis, these devices promise straightforward adaption into biological research facilities. In addition to understanding the generation of force in pathogenic species, LOC devices may also enable a better understanding of fungal/oomycete responses to environmental pollutants, such as heavy metals [80,81]. These responses have large environmental implications as fungi offer a green and sustainable means of removing heavy metals from wastewaters [82].

However, to enable a true paradigm shift, all microfluidic platforms, including micropillar systems, need to continue to simplify and standardize flow control and experimental setups to reduce complexity [83] and avoid the “chip-in-a-lab” challenge [84]. Besides the technical and usability issue that needs to be overcome, future improvements are required, especially relating to the resolution and accuracy of tracking micropillars, the automation of multiplexed measurement channels for high-throughput, and the miniaturization of measurement pillar size to extend the technology to organisms with smaller hyphae. If some of these challenges can be overcome, however, there is significant potential for the platforms reviewed here to form the foundation of key breakthroughs in our understanding of the fundamental biological processes underpinning the pathogenicity of fungal and oomycetes. This in turn promises to open up avenues for future control and treatment of plant, animal, and human diseases caused by these pathogens.

## Figures and Tables

**Table 1:** Summary of tip-growing cells monitored at the cellular and multi-cellular level.

Device Structure	Organisms	Spores/Mature Hyphae	Device Function	Refs
Mazelike micro-confined networks	Fungi: *Pycnoporus cinnabarinus*, *Neurospora crassa*	Hyphae	Testing the space searching ability of fungal hyphae	[28,29]
	Fungus: *Neurospora crassa*	Hyphae	Monitoring how constraining geometries determine the intracellular processes responsible for fungal growth	[30]
	Fungi: *Coprinellus angulatus, Psilocybe* cf. *subviscida, Gymnopus confluens, Tricholomella constricta, Leucopaxillus gentianeus, Mycetinis scorodonius, Leucoagaricus leucothites*	Hyphae	Screening different responses between species in terms of foraging range and persistence, spatial exploration, and the ability to pass obstacles	[31]
Parallel microchannels	Fungi: *Talaromyces helices*, *Neurospora crassa*	Hyphae	Monitoring the growth of hyphae under fine control of nutrient and water supply	[32]
Parallel cultivation microchamber	Fungi: *Penicillium chrysogenum*, *Fusarium virguliforme*	Spores to hyphae	Monitoring fungal morphogenesis during different stages of the life cycle	[33,36]
	Fungus: *Aspergillus ochraceus*	Spores	Monitoring germination behavior of spores for at differing pH and temperature	[34]
	Oomycete: *Phytophthora sojae*	Spores	Observing root-pathogen physicochemical interactions	[38]
	Fungus: *Coprinopsis cinerea*; Oomycete: *Pythium ultimum*	Hyphae	Analyzing the mycelial retention of phages	[39]
	Fungus: *Coprinopsis cinerea*	Hyphae	Monitoring the nutrient distribution of fungi and their defense response against fungivores nematode	[40]
Microchamber array with microvalves	Fungus: *Yarrowia lipolytica*	Yeast cells	Parallel screening of yeast cell growth and dimorphic yeast dynamics	[35]
2D Spiral Channel	Fungus: *Neurospora crassa*	Hyphae	Monitoring single-nucleus dynamics	[37]
Droplet microfluidics	Oomycete: *Phytophthora sojae*	Spores	Encapsulating single motile zoospores into each droplet, and tracking germination and germ tube growth of zoospores at different metalaxyl concentrations	[42]
	Fungus: *Aspergillus niger*	Spores	Encapsulating single zoospores, incubation, and sorting base on secreted enzyme activities for HTS	[43]
TipChip, distribution chamber access to the entrances of the microchannels	Plant: *Camellia japonica* pollen	Pollen grains	Hydrodynamically trapping pollen grains at the entrance of microchannels, and guiding pollen tube growth into channels. Monitoring germination and growth rates of pollen tubes exposed to different geometrical conditions	[44,45,46]
	Plant: *Camellia japonica* pollen	Pollen grains	Testing the Young’s modulus of the cell wall in a longitudinal direction using a bending force by fluid loading	[47]
Channel array with a single normally-open microvalve	Fungus: *Neurospora crassa*	Spores	Compartmentalizing conidia after hydrodynamic trapping. Analyzing hyphal growth in various glucose concentrations and channel geometry conditions, analyzing nuclear migration and gene expression dynamics	[50]
	Plant: *Lilium longiflorum* pollen	Pollen grains	Testing mechanical properties of pollen tubes using soft compression created by a second membrane valve	[51]
A channel array with separately controlled normally-closed microvalves	Oomycete *Achlya bisexualis*	Spores	Hydrodynamic trapping and hermetic fluidic compartmentalizing single zoospores in trap sites. Monitoring spore germination and germ tube growth	[52,53,54]
